# Trends and Predictors of Prelacteal Feeding Practices in Nigeria (2003–2013)

**DOI:** 10.3390/nu8080462

**Published:** 2016-07-29

**Authors:** Kingsley E. Agho, Pascal Ogeleka, Felix A. Ogbo, Osita K. Ezeh, John Eastwood, Andrew Page

**Affiliations:** 1 School of Science and Health, Western Sydney University, Campbelltown Campus, Locked Bag 1797, Penrith 2571, NSW, Australia; ezehosita@yahoo.com; 2 Department of Public Health, College of Science, School of Public Health, Health and Engineering La Trobe University, Bundoora 3083, VIC, Australia; ogespal@yahoo.com; 3 Centre for Health Research, School of Medicine, Western Sydney University, Campbelltown Campus, Locked Bag 1797, Penrith 2571, NSW, Australia; felgbo@yahoo.co.uk (F.A.O.); a.page@westernsydney.edu.au (A.P.); 4 Ingham Institute for Applied Medical Research, 1 Campbell Street, Liverpool 2170, NSW, Australia; John.eastwood@sswahs.nsw.gov.au; 5 Department of Community Paediatrics, Community Paediatrics, Sydney Local Health District 24 Liverpool Road, Croydon 2132, NSW, Australia

**Keywords:** prelacteal, feeding practices, Nigeria, exclusive breastfeeding

## Abstract

Prelacteal feeding practices are associated with an increased risk of diarrhoea and many early-life diseases. This paper examined trends and predictors of prelacteal feeding practices in Nigeria. A sample of 6416 infants aged 0–6 months from the Nigeria Demographic and Health Survey data for the period (2003–2013) was used. Trends and multilevel logistic regression analyses were used to determine the predictors. The trends of prelacteal feeding rates fluctuated between 55% and 66% over the study period and were significantly lower among mothers with secondary or higher levels of education (13.1%, 95% confidence interval (CI): 0.54–25.9, *p*-value = 0.041), delivered at the health facility (13.7%, CI: 1.39–25.9, *p*-value = 0.029), from more affluent households (18.7%, CI: 1.53–35.9, *p*-value = 0.033), and lived in urban areas (26.9%, CI: 18.3–35.5, *p*-value < 0.001). Multivariable analyses revealed that mothers with no schooling, younger mothers (aged 15–24 years), mothers who delivered at home, and delivered by caesarean section were more likely to introduce prelacteal feeds. Many mothers still engage in prelacteal feeding practices in Nigeria, with prelacteal feeding more prevalent in young mothers, mothers with no schooling, and mothers who delivered at home. Interventions involving community health volunteers are needed to improve feeding practices in Nigeria.

## 1. Introduction

It has been previously documented that prelacteal feeding is harmful and can expose infants to the risk of infection [1]. A recent study conducted in India indicated that infants who received prelacteal feeding were significantly more likely to be stunted and wasted compared to those who were exclusively breastfed [1]. Prelacteal feeding practice among nursing mothers deprive newborns of colostrum—rich in nutrients and immunoglobulins—thus, causing a reduction of the priming of the gastrointestinal tract, and increases the risk of infant morbidity and mortality [2,3]. Studies have shown that the type of prelacteal feeds given to the newborn is associated with the culture and belief system of the nursing mothers [4,5]. The most common prelacteal foods given to infants in many low-middle income countries could be grouped into three: water only, water-based (rice water, herbal mixture, juice), and milk-based (animal milk, infant formula) [4].

Breastmilk remains the ideal source of nutrients and natural immunity for protection of infants against infectious and chronic diseases, and development of neurocognitive systems [6,7,8,9]. Optimal breastfeeding at birth plays an important role in determining the health of a child, globally [7,8], especially those less than six months of age. In recognition of the benefits of human breastmilk, particularly when infants are exclusively breastfed, the World Health Organization (WHO) and United Nations Children’s Fund (UNICEF) recommends the avoidance of prelacteal feeding of infants within the first six months of life, if not medically indicated [4,6,10,11].

Despite significant benefits associated with exclusive breastfeeding, prelacteal feeding is still widely practiced worldwide, for example, in Asia [3,4,12,13], Latin America [14,15], and sub-Saharan African countries [2,6,16,17,18], including Nigeria. It has been suggested that insufficient flow of mother’s breastmilk, medical indications (such as prevention of dehydration or hypoglycaemia), and cultural belief systems (such as cleansing the baby’s gastrointestinal tract for digestion, quenching thirst, flushing of the bladder, and affording the mother some rest) possible reasons for why mothers practice prelacteal feeding [9,17,19]. The prevalence of prelacteal feeding in Nigeria remains the highest (56%) [10] in the sub-Saharan African region compared to other countries, like Ethiopia (45%) [2] and Uganda (31%) [6]. This could be one of the reasons for why Nigeria still has the lowest reported rate of exclusive breastfeeding in sub-Saharan Africa [20].

Previous studies on prelacteal feeding in Nigeria were hospital- and metropolitan-based studies [17,19,21], and findings from these studies may not be effective for formulating interventional policies to the wider Nigerian population because mothers who delivered at home facilities, particularly those in rural communities, were not included. The most recent report from the 2013 Nigeria Demographic and Health Survey (NDHS) indicated that nearly two-thirds of infants were born in home facilities, and majority of these births occurred in rural areas [22]. An in-depth knowledge of factors influencing introduction of prelacteal feeds is important in the promotion of exclusive breastfeeding and early initiation of breastfeeding in Nigeria [4]. Hence, this study aimed to examine trends in prelacteal feeding in Nigeria and investigate factors associated with the introduction of prelacteal feeds in Nigeria using nationally representative household survey data for the period (2003–2013). Findings from this study will provide evidence-based information to policy-makers and public health experts to inform policies and interventions that can improve infant feeding practices and child’s nutrition in Nigeria.

## 2. Materials and Methods

Data used for this study were from the 2003 (*n* = 658), 2008 (*n* = 2832) and 2013 (*n* = 2926) NDHS household surveys [8,22,23], with ethics approval from ICF International (Rockville, MD, USA). The data were used to examine the trends in prelacteal feeding, and to examine the factors associated with prelacteal feeding in Nigeria. Examining the predictors of prelacteal feeds, we pooled the three surveys. The NDHS provides information on a wide range of socio-economic, demographic, environmental, and health characteristics (including infant feeding practices) by interviewing men aged 15–59 years and women aged 15–49 years. Sampling techniques utilized in obtaining the information have been discussed in detail elsewhere [23].

In the merged dataset (*n* = 6416), the analyses used information from the most recent live newborns aged less than six months old who had prelacteal feeds within the five-year period preceding the NDHS interview date.

### 2.1. Outcome and Exploratory Variables

The key outcome variable in the study was prelacteal feeding, as reported by the mothers who were interviewed in the surveys, defined as giving any food item or liquid (except breast milk) to a newborn, within the first three days after birth [4,6,10,11]. The binary form of the outcome variable “prelacteal feeding” was noted as a “Yes” (1 = if newborn infants were given any food items or liquid within the specified period) and a “No” (0 = if newborn infants were not given any food items or liquid within the specified period). In the NDHS survey, mothers who participated were asked “in the first 3 days after delivery, was your newborn given anything to drink other than breast milk”, which was followed by 10 groups of liquid drinks, including plain water, sugar or glucose water, gripe water, sugar/salt water solution, fruit juice, milk, infant formula, tea/infusion, honey, and others.

Previous studies on prelacteal feeding [2,4,10,12,14,18], especially from low- and middle income countries, played a role in the exploratory variables selected for the study based on the data available in the pooled dataset. These variables were grouped into four classes: community level factors, socio-economic level factors, proximate determinants (maternal and newborn characteristics), and health knowledge factors. The community level factors assessed included geopolitical zone (North Central, North East, North West, South East, South West, and South South) and place of residence (rural or urban). The socio-economic level factors considered were maternal education, paternal education, maternal work status and wealth index variable which measures the economic status of men and women who participated in the survey. The proximate determinants consist of maternal and infant characteristics, maternal age at birth, and child characteristics (gender, birth place, birth order, birth interval, mode of delivery, delivery assistance, antenatal visit, and perceived newborn size by the mother). We also considered health knowledge factors consisting of the frequency of mothers listening to the radio, watching television, and reading newspapers or magazines.

The actual birth weight was not used in the study because over half of the newborns were not weighed at birth; however, perceived newborn size at birth by mothers was used as a reasonable proxy. A previous study reported that there is a close association between mean birth weight and perceived newborn size by the mother [24].

### 2.2. Statistical Analysis

Preliminary analyses involved frequency tabulations of all selected characteristics for each year of survey, followed by estimation of trends in prevalence of prelacteal feeding over a 10-year period. The Taylor series linearization method was used in the surveys when estimating 95% confidence intervals around prevalence estimates. Differences in prevalence estimates in prelacteal feeding were expressed as percentages comparing the survey across the study period. In all comparisons, differences were estimated using a chi-squared to test the significance of differences at *p* < 0.05.

Logistic regression generalized linear latent and mixed models (GLLAM) with the logit link and binomial family [25] that adjusted for cluster and survey weights were used to identify those factors associated with prelacteal feeding. A staged modelling technique was adopted for the multivariable analysis in which level-factors were entered progressively into the model to assess their relationship with the study outcome [26]. First, the community-level factors were entered into the baseline multivariable model to examine their association with the study outcome. Thereafter, a manual stepwise backwards elimination process was conducted and only variables significantly related to the study outcome at a 0.05 significance level were retained in the model (model 1). Second, socio-economic level factors were entered into model 1, and those factors with *p*-values < 0.05 were retained (model 2) after a backwards elimination process was conducted. Third, proximate determinant factors consisting of maternal and infant factors were added to model 2. As before, those factors with *p*-values < 0.05 were retained (model 3). Finally, a similar process was used for the health knowledge factors, which were entered into model 3. Once more, those factors with *p*-values < 0.05 were retained in the final model (model 4). Only those factors significantly associated with prelacteal feeding at a 5% significance level in model 4 were reported in the study.

The odds ratios (OR) and their 95% confidence interval derived from the adjusted logistic regression models were used to measure the level of association of the factors with prelacteal feeding in Nigeria. For the adjustment of the cluster sampling survey design employed in the NDHS and sampling weights, analyses were performed using “svy” commands in STATA version 13.0 (Stata Corporation, College Station, TX, USA).

## 3. Results

Of the 6416 most recent live births of infants aged less than six months used in the analyses, 3727 infants were provided with prelacteal foods. Compared with other geopolitical regions, the North West geopolitical region had the highest number of infants provided with prelacteal foods (Table 1). The number of infants provided with prelacteal foods was also higher among poor households, male infants, and those infants delivered by a non-health professional (i.e., a combination of traditional birth attendants, other unskilled worker, and no one).

### 3.1. Trends in Prelacteal Feeds

**Figure 1:**
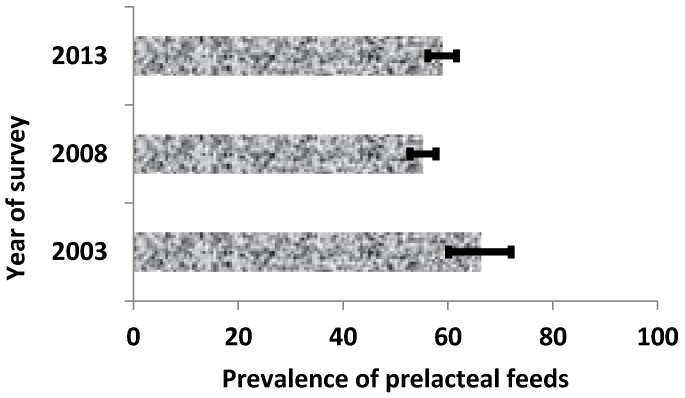
Trends in prevalence of prelacteal feeding in Nigeria (NDHS 2003–2013).

Our result indicated that the prevalence of prelacteal feeding fluctuated (i.e., between 66.4% in 2003, 55.3% in 2008, and 59.0% in 2013) over the study period (Figure 1). These results could be translated to a significant decrease of about 11% in 2008 compared to 2003, and an increase of about 4% in 2013 compared with 2008 but this increase did not differ statistically.

#### 3.1.1. Trends in Prevalence of Prelacteal Feeds by Key Factors

Table 2 shows the trends in prevalence of prelacteal feeding by key factors. The study found that the prelacteal feeding among infants aged 0–6 months of age increased significantly by 19.3% in North-Central, significantly decreased by 10.5% and 46% in North-East and South-South Geopolitical regions, respectively. The trend in prelacteal feeds reduced significantly among mothers who lived in urban areas (26.9%, *p* < 0.001). Prelacteal feeding practices among mothers who completed primary education and mothers from rich households decreased significantly by 13.1% and 18.7%, respectively over the study period.

The trend in prelacteal feeding practices decreased significantly among working mothers (9.3%, *p* = 0.027), mother who delivered their babies at the health facilities (13.7%, *p* = 0.029), mothers who delivered their infant by caesarean (43.1%, *p* < 0.001) and non-caesarean (6.9%, *p* = 0.043), mothers aged 35–49 years old (14.8%, *p* = 0.013), and the mothers delivered by health professionals (15.3%, *p* < 0.009) over the study period.

#### 3.1.2. Multivariable Analysis

Compared with other geopolitical regions, newborns born to mothers residing in the North East geopolitical region (OR = 4.77, 95% CI: 3.41–6.66) reported a significantly higher risk of prelacteal feeding. There was a significantly lower risk of prelacteal feeding for second to fourth birth order newborns (OR = 0.80, 95% CI: 0.66–0.97) compared to first birth (Table 3). A significantly higher risk of prelacteal feeding among newborns was observed if their mothers had no formal education (OR = 1.65, 95% CI: 1.33–2.03). Similar results were noted when we replaced maternal education with paternal education in the final model; that is, newborns whose fathers had no formal education were more likely to receive prelacteal feeds compared to those whose fathers had formal education (OR = 1.46, 95% CI: 1.21–1.77).

Newborns who were delivered at home were more likely to receive prelacteal feeds compared to those who were delivered at a health facility (OR = 1.45, 95% CI: 1.23–1.71). In comparison to newborns who were delivered by younger mothers (aged 15–24 years), newborns who were delivered by older mothers (aged 35–49 years) had a significantly lower risk of receiving prelacteal feeds (Table 3). Other significant factors that positively influenced prelacteal feeding in Nigeria included newborns whose deliveries were by caesarean section, and newborns whose mothers were employed (OR = 1.91, 95% CI: 1.17–3.13 and OR = 1.26, 95% CI: 1.10–1.44, respectively).

## 4. Discussion

The overall prevalence of prelacteal feeding decreased over the study period (2003–2013). A decreasing trend in the prevalence of prelacteal feeding behavior was observed among mothers with at least a primary level of education, employed mothers, older mothers aged 35–49 years. A similar decreasing trend in prelacteal feeding practice was evident among mothers who delivered their babies at a health facility compared to those who delivered at home. Mothers with no education and young mothers were more likely to engage in prelacteal feeding compared to educated and older mothers, respectively. The odds for prelacteal feeding were higher among mothers in employment and those who delivered at home compared to mothers not in employment and those who delivered at a health facility, respectively.

In interpreting the study findings, a range of methodological limitations and strengths need to be considered. First, prelacteal feeding, as an outcome in this study, was based on self-report, and this is a likely source of measurement bias, given that mothers may inaccurately recall how the child was fed at the time mothers were asked to participate in these surveys. A second possible limitation is that newborn infants who received both solid or liquid items and breast milk were not detailed in the NDHS database, and misclassification of infants who received prelacteal feeding may have occurred. However, recall bias is less likely given that analyses were restricted to the most recent birth within the five-year period preceding the surveys. Another important strength is that data used in this study were nationally representative with high response rates ranging from 95% to 98%.

The benefits of early initiation of breastfeeding within the first hour of birth and exclusive breastfeeding are well documented [4,27]. However, in many developing countries prelacteal feeding, (which is the act of giving any food item or liquid except breast milk to a newborn, within the first three days after birth, unless medically indicated) remains a common practice among new mothers [4,6,10,12,13,14,15]. Although a decreasing prevalence of prelacteal feeding was observed in the current study, this rate remains high in Nigeria compared to other sub-Saharan African countries such as Ethiopia [2], Uganda [6], and Egypt [10]. Additionally, studies from Nigeria have reported variations in the prevalence of prelacteal feeding, where socio-economic status (particularly maternal education) and the place of residence (urban or rural) played a major role [21,23]. These differences in prevalence of prelacteal feeding may reflect the population characteristics and the data source [4,21,23]. Plausible reasons for the decline in prelacteal feeding in Nigeria may be due to a decrease in the prevalence of Human Immunodeficiency Virus (HIV) infection and home delivery in Nigeria [28], as suggested by previously published studies from Uganda [29] and Bangladesh [30], respectively. In the past, the implementation of the World Health Organization’s (WHO) infant feeding recommendations for mothers living with HIV created befuddling messages that resulted in the practice of mixed feeding, including prelacteal feeding [29]. However, evidence has shown that mixed feeding increases the risk of HIV transmission compared to exclusive breastfeeding [31,32]. Thus, exclusive breastfeeding is now recommended for infants of HIV positive mothers, except when medically advised, where the infant exclusively receives infant formula. The improvement in female education and an increase in the female labor force participation—shown to improve breastfeeding practices—may also be an additional reason for the observed decrease in the prevalence of prelacteal feeding in Nigeria [33].

Geopolitical variability has been reported among Nigerian [20] and Nepalese [4] mothers in the context of suboptimal breastfeeding practices (including prelacteal feeding). In Nigeria, Islam as a religion is dominant in the northern region, while Christianity dominates in the southern region. Sub-analysis showed that prelacteal feeding practice was higher among women whose religion was Islam (17%) compared to those whose religion was Christian (10%). The northern region of Nigeria has a lower proportion of female education and poorer health care system compared to southern Nigerian [23], and this may be a reason for the geopolitical differences in prelacteal feeding in Nigeria. This further highlights the need for stronger political resolve (including community-based initiatives) to reduce prelacteal feeding behaviors of Nigerian mothers. Further, other reasons have been documented for why mothers engage in prelacteal feeding, including health inequalities, socio-cultural belief systems and religious differences [4,20,23]. Similarly, studies from Nigeria [30], Malawi [34], and Indonesia [35] have suggested that the attitude of grandmothers and traditional birth attendants (TBAs) may also be an impediment to optimal infant feeding, where grandmothers or TBAs encourage new mothers to throw away the colostrum—rich in immunoglobulins and nutrients. Community-based initiatives that consider the local environment, cultural, and religious differences of Nigerian mothers are needed to reduce prelacteal feeding practices in Nigeria.

In this study, place and mode of delivery were associated with prelacteal feeding practices. These findings were consistent with evidence from India [12,36,37,38] and Ethiopia [2], which showed that home delivery promoted prelacteal feeding. Similarly, studies from Vietnam [3], Nepal [4], and Uganda [6] found higher prevalence of prelacteal feeding among newborns whose mothers delivered by caesarean section compared to those who delivered per vaginum. Caesarean section has been found to be an obstacle to early initiation of breastfeeding in many mothers who deliver at the health facility, and this may be an additional reason for the observed finding [3,39]. In Nigeria many health care centers (particularly baby-friendly certified hospitals) promote early initiation of breastfeeding and exclusive breastfeeding [2], and also ensure provider-directed breastfeeding counselling during pregnancy, delivery, and postpartum periods to deter prelacteal feeding practices [3]. Mothers who delivered at home miss this opportunity to receive health information on optimal breastfeeding practices, and this could be a likely reason for the high prevalence of prelacteal feeding observed in mothers who delivered at home. Facility- and community-based interventions that encourage mothers to deliver in the health facility, including training of health care professionals and traditional birth attendants in Nigeria are needed to further reduce prelacteal feeding practices in Nigeria.

Consistent with evidence from Nepal [4], Uganda [6], India [12], and Honduras [40], this study found that younger maternal age was associated with prelacteal feeding compared to older mothers. A plausible explanation for this finding may be that younger mothers (likely to be primiparous) have less knowledge, skills, and experience in optimal breastfeeding practices and newborn care, and are easily influenced by the marketing of infant formulas—of increasing trend in Nigeria, mainly through the television and magazines [41,42]. The enforcement of the International Code of Marketing of Breast Milk Substitutes would also be significant in reducing prelacteal feeding practices in Nigeria.

In the present study, mothers with no formal education were more likely to engage in prelacteal feeding practices compared to those with formal education. This finding has also been reported in studies from other developing countries such Nepal [4], Uganda [6], and India [36,38]. Nonetheless, studies from Philippines [43] and China [44] found that educated mothers engaged in prelacteal feeding. Socio-cultural differences, increasing female labor force participation and changes in population demography may be the reason for the observed variation in prelacteal feeding among educated mothers.

## 5. Conclusions

The rate of prelacteal feeding in Nigeria remains one of the highest in the world. The multifactorial nature of its determinants, including socioeconomic deprivation entails the adoption of a multipronged community-based approach with special focus on the North-East and North-West regions, uneducated mothers, mothers who delivered at home, and younger and primiparous mothers, to achieve a substantial reduction in prelacteal feeding prevalence.

## Figures and Tables

**Table 1:** Characteristics of infants less than six months of age provided with prelacteal foods in Nigeria, NDHS 2003–2013.

Variables	2003 *N* (*n*)	2008 *N* (*n*)	2013 *N* (*n*)
**Community-Level Factor**			
**Geopolitical region**			
North Central	89 (32)	380 (145)	429 (235)
North East	149 (124)	496 (392)	533 (387)
North West	233 (184)	818 (549)	1020 (730)
South East	35 (11)	271 (145)	267 (135)
South West	85 (37)	393 (205)	277 (124)
South South	67 (49)	475 (128)	401 (114)
**Residence type**			
Urban	180 (132)	870 (391)	1016 (475)
Rural	478 (304)	1961 (1174)	1910 (1250)
**Socio-Economic Factors**			
**Household wealth index**			
Poor	290 (205)	975 (628)	1096 (766)
Middle	250 (161)	1034 (581)	1324 (748)
Rich	108 (64)	669 (281)	467 (189)
**Mother’s education**			
No education	333 (252)	1249 (836)	1427 (1045)
Primary	151 (88)	625 (321)	517 (268)
Secondary or higher	175 (96)	958 (407)	982 (411)
**Father’s education**			
No education	254(203)	993 (649)	1099 (829)
Primary	143 (95)	560 (317)	509 (281)
Secondary or higher	232 (126)	1150 (525)	1223 (571)
**Mother’s working status**			
Not working	293 (199)	1257 (729)	1302 (765)
Working	365 (237)	1564 (828)	1622 (958)
**Maternal and Infant Factors**			
**Mother’s age**			
15–24	264 (180)	947 (562)	970 (625)
25–34	294 (182)	1360 (709)	1427 (788)
35–49	100 (74)	524 (294)	529 (313)
**Mother’s perceived baby size**			
Small	108 (72)	465 (270)	481 (324)
Average	289 (188)	1103 (652)	1183 (740)
Large	255 (173)	1240 (635)	1251 (653)
**Sex**			
Female	313 (204)	1392 (755)	1479 (857)
Male	345 (233)	1440 (810)	1446 (868)
**Birth order**			
1	144 (98)	563 (321)	556 (319)
2 to 4	296 (180)	1271 (637)	1324 (732)
≥5	219 (159)	998 (606)	1045 (674)
**Birth interval (months)**			
No previous birth	144 (98)	563 (321)	556 (319)
<24	73 (36)	362 (191)	363 (220)
≥24	441 (303)	1900 (1051)	2003 (1184)
**Place of birth**			
Home	455 (324)	1765 (1115)	1864 (1281)
Health facility	203 (113)	1067 (450)	1062 (444)
**Mode of delivery**			
Non-caesarean	643 (427)	2780 (1535)	2843 (1688)
Caesarean	8 (6)	49 (29)	57 (28)
**Antenatal visit**			
None	263 (200)	1250 (780)	1019 (718)
1 to 3	98 (59)	344 (198)	471 (299)
≥4	295 (177)	1238 (586)	1436 (708)
**Delivery assistance**			
Health professional	215 (125)	985 (417)	1086 (466)
Traditional birth attendant	114 (82)	576 (375)	635 (438)
other unskilled worker	236 (160)	734 (437)	815 (531)
No one	93 (69)	537 (336)	390 (290)
**Health knowledge factors**			
**Reading magazine or newspaper**			
At least once a week	48 (20)	162 (57)	185 (77)
Less than once a week	57 (33)	243 (110)	225 (89)
Never	545 (376)	2344 (1366)	2497 (1544)
**Listening to radio**			
At least once a week	163 (107)	606 (320)	1024 (550)
Less than once a week	83 (43)	445 (245)	714 (385)
Never	189 (140)	996 (643)	1177 (783)
**Watching TV**			
At least once a week	62 (42)	355 (175)	828 (370)
Less than once a week	53 (27)	303 (161)	470 (224)
Never	412 (288)	1617 (1012)	1613 (1120)

*N* = Weighted number of infants < 6 months of age; *n* = Weighted number of infants provided with prelacteal foods.

**Table 2:** Prevalence and differences as percentage-points of prelacteal feeding rates by individual, household and community characteristics, Nigeria, 2003, 2008, and 2013.

Characteristic	Prelacteal Feeding Rate 2003			Prelacteal Feeding Rate 2008			Prelacteal Feeding Rate 2013		
	%	(2008–2003)	*p*	%	(2013–2008)	*p*	%	(2013–2003)	*p*
**Community-level factor**									
Geographical region									
North Central	35.5	2.8	0.704	38.3	16.50	<0.001	54.8	19.3	0.015
North East	83.1	−4.1	0.292	79.0	−6.40	0.117	72.6	−10.5	0.035
North West	78.8	−11.6	0.006	67.2	4.40	0.143	71.6	−7.2	0.087
South East	31.8	21.7	0.166	53.5	−2.80	0.609	50.7	18.9	0.233
South West	43.3	9.0	0.369	52.3	−7.40	0.086	44.9	1.6	0.872
South South	74.3	−48.3	<0.001	26.0	2.30	0.740	28.3	−46.0	<0.001
Residence type									
Urban	73.7	−28.8	<0.001	44.9	1.90	0.573	46.8	−26.9	<0.001
Rural	63.6	−3.8	0.368	59.8	5.60	0.012	65.4	1.8	0.674
**Socio-economic factors**									
Household wealth index									
Poor	70.7	−6.3	0.123	64.4	5.50	0.055	69.9	−0.8	0.833
Middle	64.3	−8.1	0.137	56.2	0.30	0.916	56.5	−7.8	0.151
Rich	59.2	−17.1	0.046	42.1	−1.60	0.679	40.5	−18.7	0.033
Mother’s education									
No education	75.9	−9.0	0.017	66.9	6.40	0.010	73.3	−2.6	0.493
Primary	58.4	−7.1	0.248	51.3	0.60	0.885	51.9	−6.5	0.292
Secondary or higher	55.1	−12.5	0.052	42.6	−0.60	0.815	42.0	−13.1	0.041
Father’s education									
No education	79.9	−14.7	<0.001	65.2	10.30	<0.001	75.5	−4.4	0.219
Primary	66.6	−9.9	0.097	56.7	−1.50	0.701	55.2	−11.4	0.06
Secondary or higher	54.5	−8.8	0.073	45.7	1.00	0.671	46.7	−7.8	0.115
Mother’s working status									
Not working	68.1	−10.1	0.013	58.0	0.80	0.748	58.8	−9.3	0.027
Working	65.0	−12.0	0.004	53.0	6.10	0.013	59.1	−5.9	0.157
**Maternal and infant factors**									
Mother’s age									
15–24	68.2	−8.9	0.098	59.3	5.10	0.062	64.4	−3.8	0.489
25–34	62.2	−10.0	0.017	52.2	3.00	0.224	55.2	−7.0	0.109
35–49	73.9	−17.9	0.002	56.0	3.10	0.388	59.1	−14.8	0.013
Mother’s perceived baby size									
Small	66.4	−8.2	0.277	58.2	9.20	0.013	67.4	1.0	0.901
Average	64.9	−5.8	0.144	59.1	3.50	0.180	62.6	−2.3	0.551
Large	67.9	−16.7	<0.001	51.2	1.00	0.691	52.2	−15.7	0.001
Sex									
Female	65.2	−11.0	0.004	54.2	3.70	0.119	57.9	−7.3	0.069
Male	67.4	−11.2	0.014	56.2	3.80	0.103	60.0	−7.4	0.103
Birth order									
1	68.0	−10.9	0.148	57.1	0.30	0.941	57.4	−10.6	0.166
2 to 4	60.7	−10.6	0.024	50.1	5.20	0.045	55.3	−5.4	0.260
≥5	72.9	−12.1	0.005	60.8	3.70	0.161	64.5	−8.4	0.058
Birth interval (months)									
No previous birth	68.0	−10.9	0.148	57.1	0.30	0.941	57.4	−10.6	0.166
<24	49.7	3.0	0.768	52.7	7.40	0.078	60.1	10.4	0.287
≥24	68.6	−13.2	<0.001	55.4	3.70	0.084	59.1	−9.5	0.009
Place of birth									
Home	71.2	−8.0	0.022	63.2	5.50	0.008	68.7	−2.5	0.478
Health facility	55.5	−13.3	0.032	42.2	−0.40	0.904	41.8	−13.7	0.029
Mode of delivery									
Non-caesarean	66.3	−11.1	0.001	55.2	4.20	0.026	59.4	−6.9	0.043
Caesarean	92.7	−34.4	0.002	58.3	−8.70	0.438	49.6	−43.1	<0.001
Antenatal visit									
None	76.1	−13.7	0.001	62.4	8.10	0.003	70.5	−5.6	0.181
1 to 3	60.6	−2.8	0.721	57.8	5.60	0.182	63.4	2.8	0.713
≥4	60.1	−12.8	0.009	47.3	2.00	0.42	49.3	−10.8	0.026
Delivery assistance									
Health professional	58.2	−15.9	0.007	42.3	0.60	0.828	42.9	−15.3	0.009
Traditional birth attendant	71.7	−6.5	0.319	65.2	3.80	0.291	69.0	−2.7	0.676
other unskilled worker	68.0	−8.5	0.075	59.5	5.70	0.069	65.2	−2.8	0.559
No one	74.5	−12.0	0.053	62.5	11.80	0.001	74.3	−0.2	0.984
**Health knowledge factors**									
Reading magazine or newspaper									
At least once a week	41.3	−6.0	0.649	35.3	6.50	0.285	41.8	0.5	0.968
Less than once a week	58.4	−13.2	0.142	45.2	−5.80	0.301	39.4	−19.0	0.036
Never	69.0	−10.7	0.001	58.3	3.50	0.073	61.8	−7.2	0.026
Listening to radio									
At least once a week	65.7	−12.8	0.018	52.9	0.90	0.788	53.8	−11.9	0.024
Less than once a week	52.4	2.8	0.702	55.2	−1.20	0.743	54.0	1.6	0.824
Never	74.2	−9.7	0.031	64.5	2.00	0.481	66.5	−7.7	0.098
Watching TV									
At least once a week	68.1	−18.7	0.034	49.4	−4.70	0.248	44.7	−23.4	0.006
Less than once a week	51.5	1.7	0.869	53.2	−5.60	0.193	47.6	−3.9	0.701
Never	70.0	−7.4	0.055	62.6	6.90	0.003	69.5	−0.5	0.887

**Table 3:** Adjusted and unadjusted odd ratios (95% confidence interval (CI)) for factors associated with prelacteal feeding in Nigeria, (NDHS, 2003–2013).

Variables	Unadjusted	Adjusted ^^,^*
	OR (95% CI)	OR (95% CI)
**Year of survey**		
2003	*Ref*	*Ref*
2008	0.63 (0.47–0.83)	0.72 (0.54–0.97)
2013	0.73 (0.54–0.98)	0.79 (0.58–1.06)
**Geographical region**		
South South	*Ref*	*Ref*
North East	7.34 (5.41–9.97)	4.77 (3.41–6.66)
North West	5.38 (4.21–6.89)	3.31 (2.50–4.39)
South East	2.32 (1.71–3.14)	2.53 (1.83–3.48)
South West	2.11 (1.61–2.78)	1.98 (1.48–2.65)
North Central	1.90 (1.43–2.52)	1.47 (1.11–1.95)
**Residence type**		
Urban	*Ref*	
Rural	1.80 (1.54–2.10)	
**Household wealth index**		
Poor	*Ref*	
Middle	0.64 (0.55–0.74)	
Rich	0.36 (0.30–0.43)	
**Mother’s education**		
Secondary or higher	*Ref*	*Ref*
Primary	1.44 (1.22–1.71)	1.13 (0.94–1.37)
No education	3.20 (2.72–3.75)	1.65 (1.33–2.03)
**Father’s education**		
Secondary or higher	*Ref*	
Primary	1.51 (1.28–1.78)	
No education	2.86 (2.45–3.35)	
**Mother’s working status**		
Not working	*Ref*	*Ref*
Working	0.91 (0.80–1.03)	1.26 (1.10–1.44)
**Mother’s age**		
35–49	*Ref*	*Ref*
15–24	1.17 (0.99–1.38)	1.31 (1.04–1.64)
25–34	0.83 (0.71–0.97)	1.02 (0.86–1.21)
**Mother’s perceived baby size**		
Small	*Ref*	
Average	0.92 (0.78–1.10)	
Large	0.66 (0.55–0.79)	
**Sex**		
Male	*Ref*	
Female	0.92 (0.82–1.03)	
**Birth order**		
2 to 4	*Ref*	*Ref*
1 to 3	1.22 (1.03–1.44)	1.25 (1.03–1.52)
≥5	1.52 (1.33–1.73)	1.18 (1.00–1.39)
**Birth interval (months)**		
No previous birth	*Ref*	
<24	0.90 (0.72–1.14)	
≥24	1.00 (0.85–1.17)	
**Place of birth**		
Health facility	*Ref*	*Ref*
Home	2.62 (2.29–3.01)	1.45 (1.23–1.71)
**Mode of delivery**		
Non-caesarean	*Ref*	*Ref*
Caesarean	0.91 (0.58–1.43)	1.91 (1.17–3.13)
**Antenatal visit**		
None	*Ref*	
1 to 3	0.77 (0.63–0.94)	
≥4	0.48 (0.42–0.56)	
**Delivery assistance**		
Health professional	*Ref*	
Traditional birth attendance (TBA)	2.64 (2.19–3.19)	
other unskilled worker	2.18 (1.86–2.55)	
No one	2.71 (2.23–3.30)	
**Reading magazine or newspaper**		
At least once a week	*Ref*	
Less than once a week	1.23 (0.89–1.70)	
Never	2.43 (1.88–3.15)	
**Listening to radio**		
At least once a week	*Ref*	
Less than once a week	0.99 (0.83–1.19)	
Never	1.64 (1.39–1.93)	
**Watching television**		
At least once a week	*Ref*	
Less than once a week	1.12 (0.90–1.38)	
Never	2.22 (1.87–2.64)	

^: Independent variables adjusted for: year of survey, geopolitical zone, place of residence, wealth index, mother’s (education, work status, age), father’s education, child’s gender, perceived baby size by mother’s, birth order, birth interval, place of birth, mode of delivery, antenatal visit, delivery assistance, reading magazine or newspaper, listening to radio, and watching television. *: Missing values were not included in the analysis.
